# Supplementary material for: Altered perivascular diffusivity in glioblastoma: integrating DTI-ALPS index with radio-pathomic and histopathologic correlates
Source: J Neurooncol. 2026 Feb 21;177(1):12. doi: 10.1007/s11060-026-05458-x (PMC12924826; doi:10.1007/s11060-026-05458-x)
Supplement: Supplementary file 1 — Supplementary Material 1 [file 11060_2026_5458_MOESM1_ESM.docx]

**Supplementary Methods**

M1. Impact of Spatial Normalization on DTI-ALPS

To assess the impact of spatial normalization on DTI-ALPS estimation, ALPS indices were computed in a subset of 100 subjects using two complementary approaches: (i) template-space estimation using diffusion tensor maps after nonlinear normalization to the JHU-ICBM-FA template, and (ii) native-space estimation using template-defined ALPS ROIs inverse-warped to each subject’s native diffusion space. For each subject, FA images were registered to the JHU-ICBM-FA template using Advanced Normalization Tools (ANTs), yielding subject-specific affine and nonlinear deformation fields and their inverses. These transforms were applied to the diffusivity maps to generate template-space images using linear interpolation, while ALPS ROIs defined in JHU space were inverse-transformed to native space using nearest-neighbor interpolation. DTI-ALPS indices were computed using identical hemisphere-specific ROI definitions in both spaces. Agreement between native- and template-space ALPS estimates was assessed by evaluating relative differences between the two measurements.

M2. Statistical Modelling for Multivariate linear regression

Associations between DTI-ALPS metrics and imaging-derived tumor features were evaluated using multivariable linear regression, with ALPS (mean or ipsilateral) as the dependent variable. Imaging predictors included contrast-enhancing (CE) volume, FLAIR hyperintensity volume, CE and FLAIR cellularity, and mean fractional anisotropy (FA) within CE and FLAIR regions. Tumor volume and cellularity measures were log-transformed, and all imaging predictors were standardized prior to analysis (z-score). Models were adjusted for age, sex, extent of resection (GTR/STR), and tumor location. Regression coefficients were estimated using ordinary least squares.

Model Specification

For subject *i*, the general model was defined as:

$$\text{ALPS}_{i}=\beta_{0}+\beta_{1}X_{i}+\sum_{k=2}^{K} \beta_{k}C_{ik}+\varepsilon_{i}$$

where:

ALPS*_i_* denotes DTI-ALPS metric for the subject *i*,

X*_i_* is the primary imaging predictor of interest,

C*_ik_* represents covariates included to control for confounding,

β*_o_* is the intercept,

β*_k_* is the regression coefficients

ε*_i_* is the error term.

M3. Survival Analysis

Overall survival was evaluated using multivariable Cox proportional hazards regression, with DTI-ALPS modeled as a standardized continuous variable and adjustment for age, sex, extent of resection, and tumor location. Kaplan–Meier survival curves stratified by median DTI-ALPS were generated for unadjusted visualization, with group differences assessed using the log-rank test.

Mathematical formulation

$$h_{i}\left( t \right)=h_{0}\left( t \right)\exp\left( \beta_{1}\text{ALP}\text{S}_{\text{i}}^{\text{*}}+\sum_{k} \beta_{k}C_{ik} \right)$$

where;

H_i_(t) denotes the hazard at time t for subject i,

H_o_(t) is the baseline hazard function,

ALPS_i_^*^ is the standardized DTI-ALPS metric,

C*_ik_* denotes the covariates

β_1_ and β_k_ are the regression coefficients.

M3. Classification of deep and lobular tumor

Tumor location was classified as deep versus lobar using contrast-enhancing tumor segmentations spatially normalized to JHU template space. A binary deep white matter mask was derived from the JHU ICBM white matter label atlas (<https://identifiers.org/neurovault.image:1401>).The correspondence between atlas labels and white matter tracts is provided in Supplementary Table T4. For each subject, the contrast-enhancing tumor segmentation was binarized in template space. The number of tumor voxels overlapping the deep white matter mask was computed, and tumors were classified as deep if the overlap fraction was ≥1%; all others were classified as lobar. All the classifications were further manually reviewed by board certified radiologist.

M4. Tumor laterality and midline crossing

Tumor laterality was determined from binary tumor masks (CE and FLAIR) in their native space. The mid-sagittal plane was operationally defined as the midpoint of the left–right voxel dimension of the template-space mask grid. Tumor voxels were counted separately in the left and right hemispheres. Laterality was assigned to the hemisphere containing the majority of tumor voxels, and a laterality fraction was computed as the proportion of voxels in the dominant hemisphere. Midline crossing was defined as the presence of tumor voxels in both hemispheres. The computational procedure is summarized in supplementary figure S2.

Supplementary Figures

**Supplementary Figure S1**. Agreement between native-space and template-space DTI-ALPS measurements.


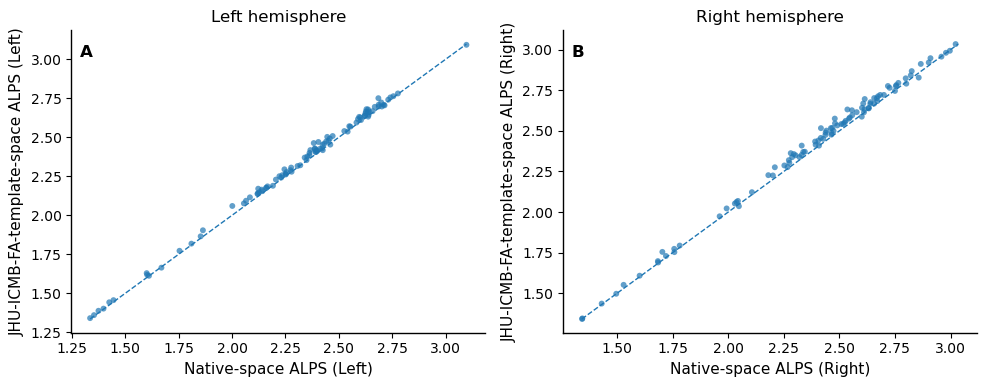


**Figure S1**. Scatter plots show native versus JHU-ICBM-FA template ALPS indices for the left (A) and right (B) hemispheres. Across 100 subjects, the median native-to-template ALPS ratio was 0.99 for both hemispheres (left IQR: 0.99–1.00; right IQR: 0.98–0.99). All measurements fell within ±5% of unity.


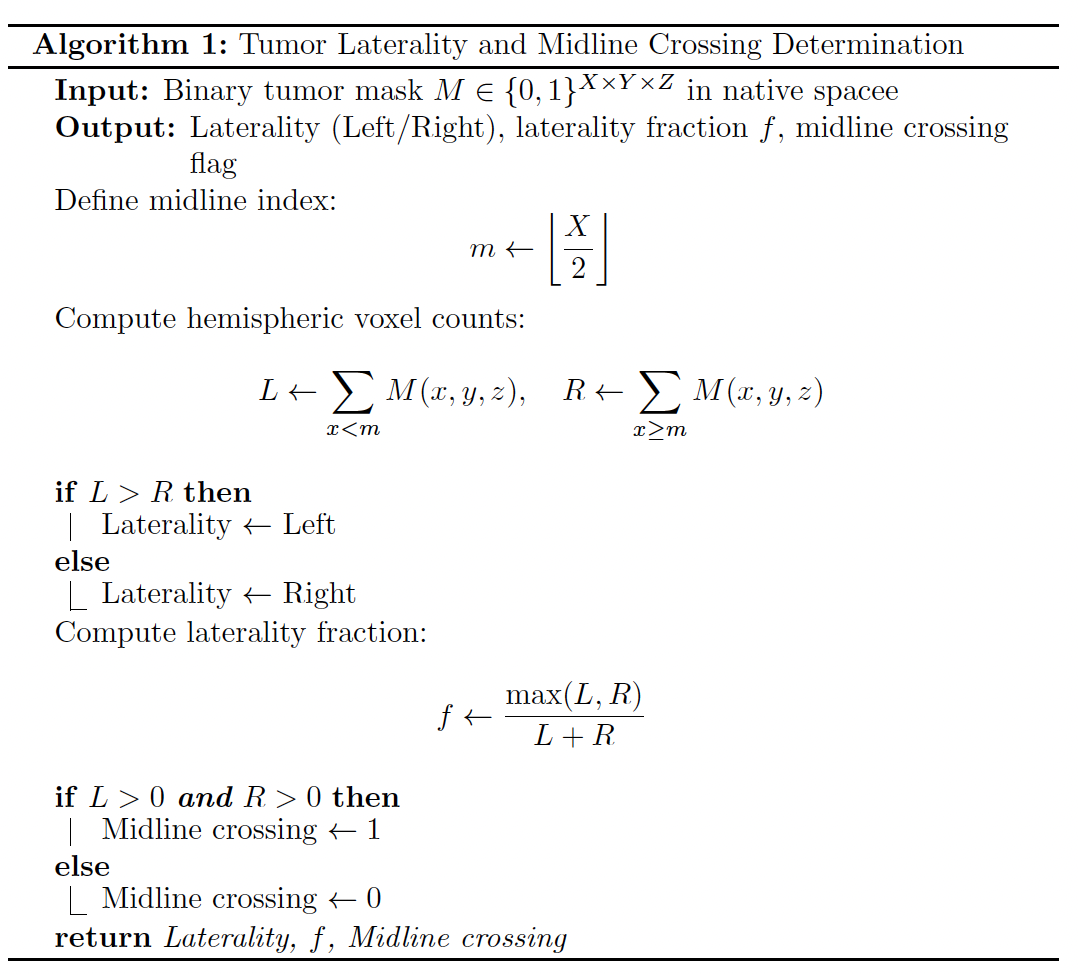


**Figure S2**. Pseudocode describing the computation of tumor laterality and midline crossing from template-space tumor masks.

**Figure S3.** Perivascular tumor invasion in glioblastoma autopsy tissue in 12 patients assessed at autopsy with SOX2 IHC. Representative images show SOX2 expressing cells infiltrating brain parenchyma and tracking along perivascular spaces in glioblastomas.. Patient demographics and treatment information for the illustrated cases are provided in ***Supplementary Table (T3).***

**
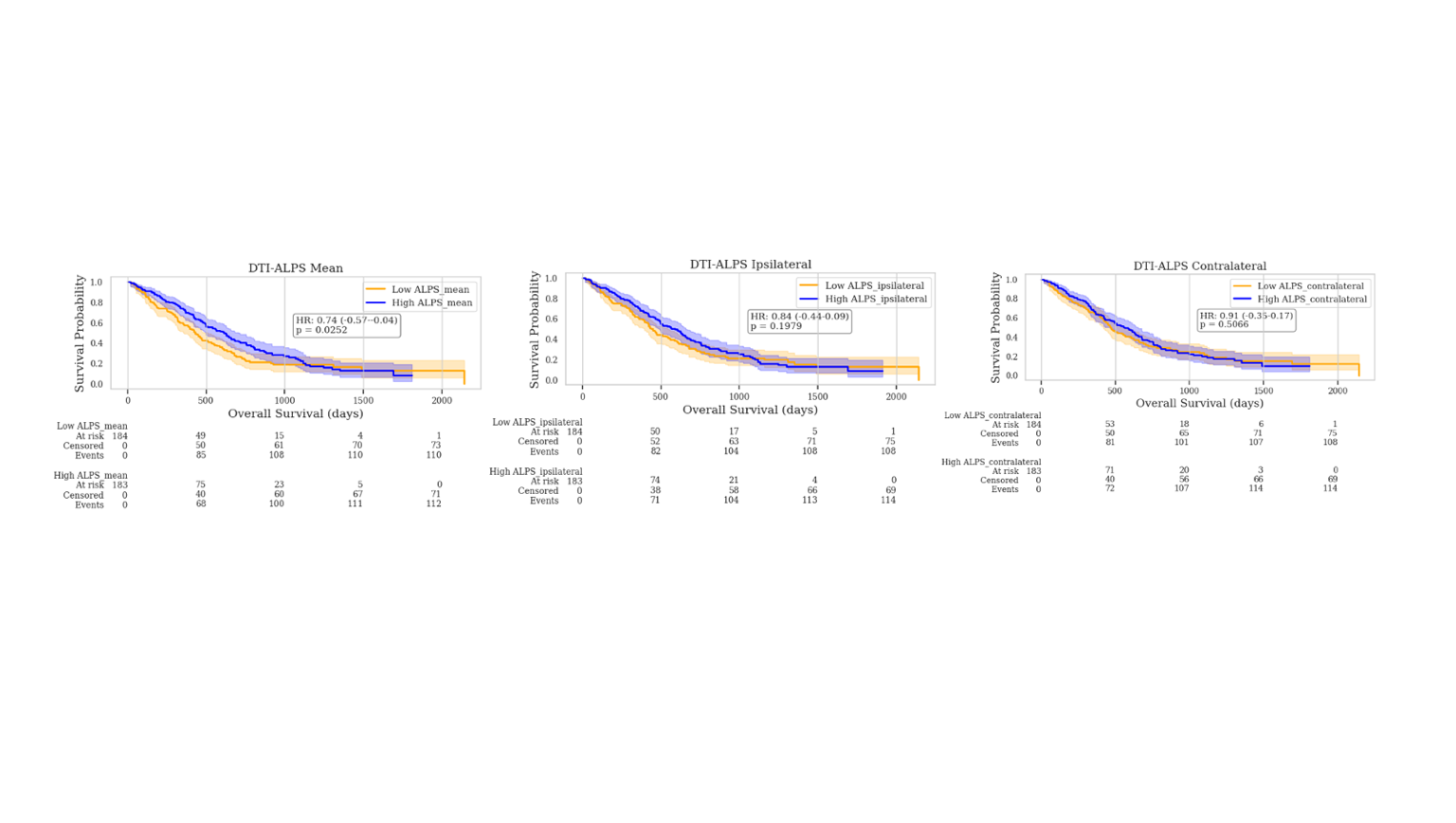
**

**Figure S4.** Overall survival stratified by DTI-ALPS indices.

Kaplan–Meier survival curves show overall survival probability for patients grouped by median split into high vs. low DTI-ALPS values for (left) DTI-ALPS_mean_, (middle) DTI-ALPS _ipsilateral,_and (right) DTI-ALPS_contralateral_.

**Supplementary Table**

T1. Multivariable linear regression models assessing associations between DTI-ALPS and imaging-derived tumor features

| DTI-ALPS Metric | Imaging Predictor | Standardized β (95% CI) | p-value |
| --- | --- | --- | --- |
| ALPS _mean_ | log (CE volume) | −0.056 (−0.083, −0.028) | 7.4×10⁻⁵ |
|  | log (FLAIR volume) | −0.056 (−0.083, −0.028) | 7.4×10⁻⁵ |
|  | log (CE cellularity) | −0.051 (−0.079, −0.023) | 3.4×10⁻⁴ |
|  | log (FLAIR cellularity) | −0.063 (−0.106, −0.020) | 0.0037 |
|  | FA in CE region | 0.050 (0.026, 0.074) | 5.4×10⁻⁵ |
|  | FA in FLAIR region | 0.066 (0.039, 0.093) | 2.0×10⁻⁶ |
| ALPS _ipsilateral_ | log (CE volume) | −0.146 (−0.191, −0.102) | 1.2×10⁻¹⁰ |
|  | log (FLAIR volume) | −0.146 (−0.191, −0.102) | 1.2×10⁻¹⁰ |
|  | log (CE cellularity) | −0.141 (−0.186, −0.095) | 1.3×10⁻⁹ |
|  | log (FLAIR cellularity) | −0.150 (−0.237, −0.063) | 7.6×10⁻⁴ |
|  | FA in CE region | 0.078 (0.037, 0.118) | 1.8×10⁻⁴ |
|  | FA in FLAIR region | 0.091 (0.047, 0.134) | 4.6×10⁻⁵ |

Supplementary Table T1. Multivariable linear regression analyses evaluating associations between DTI-ALPS metrics and imaging-derived tumor features. Reported values correspond to standardized regression coefficients (β) for the primary imaging predictor, with 95% confidence intervals. All models were adjusted for age at MRI, sex, extent of resection (GTR vs STR), and tumor location (deep vs lobar). Tumor volumes and cellularity measures were log-transformed prior to analysis.

T2. Clinical, demographic, and imaging characteristics stratified by tumor location

| **Characteristic** | **Overall (N=368)** | **Lobular** | **Deep/eloquent** |
| --- | --- | --- | --- |
| N | 368 | 84 | 284 |
| Age (years), mean ± SD | 61.8 ± 12.0 | 61.2 ± 11.1 | 62.0 ± 12.3 |
| Male, n (%) | 219 (59.5%) | 51 (60.7%) | 168 (59.2%) |
| Female, n (%) | 149 (40.5%) | 33 (39.3%) | 116 (40.8%) |
| Left hemisphere, n (%) | 194 (52.7%) | 59 (70.2%) | 135 (47.5%) |
| Right hemisphere, n (%) | 174 (47.3%) | 25 (29.8%) | 149 (52.5%) |
| Deep location, n (%) | 284 (77.2%) | 0 (0.0%) | 284 (100.0%) |
| Contrast-enhancing volume (mm³), median [IQR] | 18973 [9134–31493] | 7829 [4791–11711] | 23724 [13582–35404] |
| FLAIR hyperintensity volume (mm³), median [IQR] | 18973 [9134–31493] | 7829 [4791–11711] | 23724 [13582–35404] |
| EOR: GTR, n (%) | 214 (58.2%) | 64 (76.2%) | 150 (52.8%) |
| EOR: STR, n (%) | 112 (30.4%) | 12 (14.3%) | 100 (35.2%) |
| EOR: biopsy, n (%) | 41 (11.1%) | 8 (9.5%) | 33 (11.6%) |
| EOR: unavailable, n (%) | 1 (0.3%) | 0 (0.0%) | 1 (0.4%) |
| MGMT: positive, n (%) | 247 (67.1%) | 61 (72.6%) | 186 (65.5%) |
| MGMT: negative, n (%) | 104 (28.3%) | 20 (23.8%) | 84 (29.6%) |
| MGMT: indeterminate, n (%) | 5 (1.4%) | 0 (0.0%) | 5 (1.8%) |
| MGMT: unavailable, n (%) | 12 (3.3%) | 3 (3.6%) | 9 (3.2%) |

Data are presented as mean ± SD, median [IQR], or n (%). EOR extent of resection; GTR = gross total resection; STR = subtotal resection. Deep-seated tumors were defined by overlap of CE tumor mask with deep/eloquent white matter tracts in JHU-ICMB space.

**Table T3.** Clinical and molecular characteristics of glioblastoma patients with available autopsy tissue

| Patient ID | Age | Sex | IDH status | Overall Survival (days) | Treatment/s |
| --- | --- | --- | --- | --- | --- |
| 133 | 62 | F | WT | 273 | RT, TMZ, VMAT, Bev |
| 140 | 55 | M | WT | 538 | TMZ, TT field, VMAT, Bev |
| 142 | 41 | M | WT | 352 | TT, TMZ, XRT, CCNU |
| 146 | 54 | M | WT | 386 | Bev, XRT, ILDR |
| 170 | 48 | M | WT | 148 | **Untreated** |
| 179 | 51 | M | Mutant | 912 | Bev, TMZ, IMRT |
| 230 | 76 | M | WT | 25 | Untreated |
| 236 | 48 | M | Mutant | 781 | TT, Bev, TMZ, XRT |
| 242 | 58 | M | WT | 359 | Bev, TT, TMZ |
| 245 | 83 | M | WT | 77 | **Untreated** |
| 228 | 66 | M | WT | 127 | TMZ, IMRT |
| 209 | 65 | F | WT | 823 | TMZ, IMRT |

**Table T3.** Demographic, molecular, survival, and treatment information for glioblastoma patients with available autopsy tissue from the MCW neurooncology brain bank.

| **Tract Class** | **JHU-ICMB Label** | **White-Matter Tract** |
| --- | --- | --- |
| Commissural pathways | 4 | Genu of corpus callosum |
|  | 5 | Body of corpus callosum |
|  | 6 | Splenium of corpus callosum |
| Projection pathways | 16 | Cerebral peduncle (right) |
|  | 17 | Cerebral peduncle (left) |
|  | 18 | Anterior limb of internal capsule (right) |
|  | 19 | Anterior limb of internal capsule (left) |
|  | 20 | Posterior limb of internal capsule (right) |
|  | 21 | Posterior limb of internal capsule (left) |
|  | 22 | Retrolenticular internal capsule (right) |
|  | 23 | Retrolenticular internal capsule (left) |
| Periventricular projection / association pathways | 24 | Anterior corona radiata (right) |
|  | 25 | Anterior corona radiata (left) |
|  | 26 | Superior corona radiata (right) |
|  | 27 | Superior corona radiata (left) |
|  | 28 | Posterior corona radiata (right) |
|  | 29 | Posterior corona radiata (left) |
|  | 30 | Posterior thalamic radiation |
|  | 31 | Posterior thalamic radiation |
|  | 32 | Sagittal stratum |
|  | 33 | Sagittal stratum |
| Limbic | 7 | Fornix |
|  | 40 | Fornix |
|  | 41 | Fornix |
| Brainstem | 2 | Middle cerebellar peduncle |
|  | 3 | Pontine crossing tract |

**Table T4:** White-matter tracts used to define deep / eloquent tumor location (JHU ICBM atlas)
